# Review of CNN-Based Approaches for Preprocessing, Segmentation and Classification of Knee Osteoarthritis

**DOI:** 10.3390/diagnostics16030461

**Published:** 2026-02-02

**Authors:** Sudesh Rani, Akash Rout, Priyanka Soni, Mayank Gupta, Naresh Kumar, Karan Kumar

**Affiliations:** 1 Computer Science and Engineering Department, Punjab Engineering College, Chandigarh 160012, India; sudeshrani@pec.edu.in (S.R.); akashrout.bt21cse@pec.edu.in (A.R.); priyankasoni.bt21cse@pec.edu.in (P.S.); mayankgupta@pec.edu.in (M.G.); 2 Department of Computer Science, Birkat Al Mouz, University of Nizwa, Nizwa 616, Oman; 3 Maharishi Markandeshwar Engineering College, Maharishi Markandeshwar (Deemed to be University), Mullana 133207, India; karan.170987@gmail.com

**Keywords:** deep learning, osteoarthritis, knee, neural networks, computer, osteoarthritis classification, radiographic image interpretation, computer-assisted

## Abstract

Osteoarthritis (OA) is a prevalent joint disorder characterized by symptoms such as pain and stiffness, often leading to loss of function and disability. Knee osteoarthritis (KOA) represents the most prevalent type of osteoarthritis. KOA is usually detected using X-ray radiographs of the knee; however, the classification of disease severity remains subjective and varies among clinicians, motivating the need for automated assessment methods. In recent years, deep learning–based approaches have shown promising performance for KOA classification tasks, particularly when applied to structured imaging datasets. This review analyzes convolution neural network (CNN)-based approaches reported in the literature and compares their performance across multiple criteria. Studies were identified through systematic searches of IEEE Xplore, SpringerLink, Elsevier (ScienceDirect), Wiley Online Library, ACM Digital Library, and other sources such as PubMed and arXiv, with the last search conducted in March 2025. The review examines datasets used (primarily X-ray and MRI), preprocessing strategies, segmentation techniques, and deep learning architectures. Reported classification accuracies range from 61% to 98%, depending on the dataset, imaging modality, and task formulation. Finally, this paper highlights key methodological limitations in existing studies and outlines future research directions to improve the robustness and clinical applicability of deep learning–based KOA classification systems.

## 1. Introduction

Osteoarthritis (OA) is a chronic joint disorder marked by the progressive deterioration of cartilage in the joints. OA breaks down the cartilage and wears it away, leading to pain, stiffness, swelling, and reduced joint movement [1]. Knee OA (KOA) affects the knee joint, leading to pain and limitations in knee movement for those affected. Many factors, such as age, obesity, injury, joint overuse, and genetics, etc., contribute to the development and progression of KOA. This disease has a high prevalence among older people and causes severe discomfort and restriction in movement. The number of people with KOA has increased in recent decades [2] and is also projected to increase in the future [3]. The diagnosis and treatment of this disease also create an economic burden on individuals and society as a whole. Multiple studies have shown that early detection and treatment reduce the effect of KOA to a large extent. Moreover, early detection also reduces the cost of treatment exponentially [4]. The diagnostic accuracy of clinical evaluations and imaging investigations provided by healthcare providers is highly dependent on the knowledge and experience of doctors and therefore poses the risk of misclassification by amateur physicians [5]. Orthopedists, or specialists in joints, have the knowledge, testing abilities, and experience to diagnose; however, they face an ever-increasing workload with rising KOA cases. Additionally, the classification criteria are highly subjective, and different doctors or the same doctor at different times may have different diagnoses on the same X-ray [6]. Hence, there is a need for an automated standardized technique for the detection and classification of KOA at early stages. Such techniques and solutions can help improve the lives of millions of people every year and reduce the burden on the healthcare system.

### Significance of This Review

A detailed review of the extended literature ensures the importance of this article, specifically in the progress of deep learning (DL) architectures. Multiple researchers and experts have proposed various approaches to address the need for automated techniques using machine learning (ML) and DL algorithms to detect and classify KOA. This paper presents an extensive survey of existing techniques for automatic detection and classification of KOA. The paper also highlights the limitations of the existing methods and outlines possible future research directions in this area. This paper offers a comparative review of current methods for estimating minimal joint space width and assessing KOA severity using the Kellgren and Lawrence (KL) grading system. The paper also compares the recently proposed DL techniques based on their experimental results.

As shown in Table 1, it is evident that previous recent review papers lack the detailed analysis of various prominent preprocessing techniques and segmentation methods along with dataset details, especially MRI datasets for KOA classification. Therefore, the major research contributions of the paper are as follows:

(a) A comprehensive survey of relevant recent research studies is carried out, exploring various data sources, data preprocessing techniques, and DL architectures utilized.
(b) A comparison of performance measures of the research studies is presented. Also, the effect of variations in methodologies on the performance measures such as accuracy, precision, recall, and F1-score, etc., is discussed.
(c) Shortcomings of the considered research studies are analyzed, and promising future research directions are outlined.
(d) The review of different preprocessing methods is added as shown in Table 1.

**Table 1:** Comparison of survey papers with the proposed review in terms of preprocessing, segmentation, deep learning techniques, and imaging dataset coverage.

Paper	Year	Preprocessing Techniques	Segmentation Techniques	DL Techniques	X-ray Dataset	MRI Dataset
Kokkotis et al. [7]	2020		✓	✓	✓	✓
Saini et al. [4]	2021		✓	✓	✓	
Yeoh et al. [8]	2021		✓	✓	✓	✓
Yick et al. [9]	2022		✓	✓	✓	✓
Lee et al. [10]	2022			✓	✓	
Ramazanian et al. [11]	2023				✓	✓
Cigdem et al. [12]	2023		✓	✓	✓	✓
Zhao et al. [13]	2024			✓	✓	
Touahema et al. [14]	2024		✓	✓	✓	
Teoh et al. [15]	2024			✓	✓	✓
Tariq et al. [16]	2025			✓	✓	✓
This Review		✓	✓	✓	✓	✓

This paper is organized in various sections as follows: Section 2 explains the causes, symptoms, and prevalence of KOA along with its classification criteria. Section 3 lists the sources, selection criteria, and overall process of the literature review. Section 4 highlights the increased use of DL in healthcare and discusses the prominent DL architectures in this application domain. Section 5 elaborates on different data sources used in the papers considered for the review. Various DL approaches used in these research studies, along with data augmentation, data preprocessing, and segmentation techniques, are analyzed in Section 6. Section 7 presents the possible future research directions based on the analyzed research gaps. Lastly, the findings of the review paper are concluded in Section 8.

## 2. Osteoarthritis Overview

**Figure 1:**
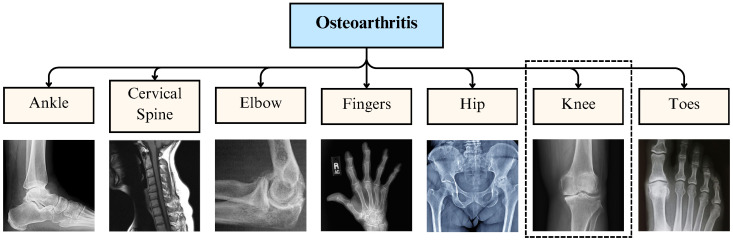
Commonly affected joints in osteoarthritis.

OA is a degenerative joint disorder caused by the deterioration of joint cartilage and the bone beneath it. It is one of the leading causes of disability in the world [17]. The most common symptoms are joint pain and stiffness, and the symptoms usually progress slowly over the years. Other symptoms include joint swelling, reduced range of motion, malfunctioning gait, and weakness or numbness among arms and legs. OA is the leading source of physical disability and impaired quality of life in industrialized nations and is expected to rise with the continuous industrial expansion and aging population [18]. The radiographic features conventionally used to define OA include joint space narrowing, osteophytes, subchondral sclerosis, cyst formation, and abnormalities of bone contour [1]. The principal morphological characteristic of OA is a slowly developing degenerative breakdown of cartilage with only episodic synovitis. The other characteristics include changes occurring in the bone, the synovium, and the muscles [19]. The hallmark of OA pathology is the loss of articular cartilage, which is commonly detected on standard radiographs as a narrowing of the joint space [20]. Osteoarthritis is the most common degenerative joint disorder that affects one or several diarthrodial joints, including small joints (such as those in the hand) and large joints (such as the knee and hip joints) [21]. Effects of OA can be observed on any joint in the body; however, most commonly affected joints are displayed in Figure 1.

KOA is the most prevalent type of OA. The following subsections explain the biological symptoms exhibited by osteoarthritis in the knee, existing medical identification techniques for KOA, its severity grading criteria, and how DL can assist in the automated detection and classification of KOA.

### 2.1. Knee Osteoarthritis

**Figure 2:**
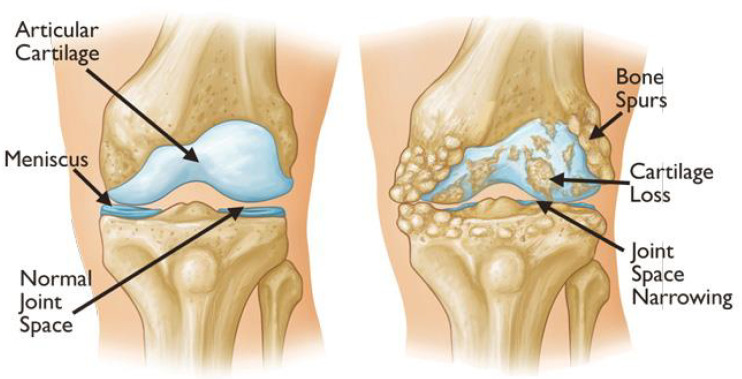
Pathological changes in an osteoarthritis-affected knee, including bone spurs, cartilage degradation, and narrowing of the joint space [24].

KOA is the category of osteoarthritis that involves the knee joint and leads to its deformity. According to [22], knee osteoarthritis (KOA) is not just a cartilage-specific condition but is viewed as a chronic disorder affecting the entire joint, including the articular cartilage, meniscus, ligaments, and surrounding muscles, which may arise from various pathophysiological processes. According to [23], knee osteoarthritis can lead to symptoms such as crepitus, bone enlargement, decreased knee flexion, flexion contracture, and tenderness. The bone spurs, cartilage loss, and joint space narrowing are depicted in Figure 2. KOA is identified by symptoms such as joint pain and functional impairments in the knee, which can disrupt both work and daily activities for patients. The KOA adversely affects the functional independence of the patient and negatively impacts the patient’s lifestyle apart from causing pain and disability. Consequently, KOA can lead to persistent pain, crepitus, swelling, morning stiffness, muscle atrophy, reduced quadriceps strength, and impaired postural control.

KOA is broadly of two types, namely patellofemoral osteoarthritis and tibiofemoral osteoarthritis. Patellofemoral OA occurs due to the loss of cartilage of the patella and the trochlear groove. It contributes towards 40% (approx.) of the overall KOA cases [25]. At the same time, tibiofemoral OA occurs due to bone spur formation in the medial tibiofemoral compartment and causes the remaining 60% of KOA cases [26]. Around 30% of people over the age of 60 suffer from KOA across the globe [22] and it is one of the major causes of impairment among the elderly population. Moreover, the cases of KOA have been continuously rising over the past few decades [2]. The authors of [27] identify aging as one of the major reasons for the development of KOA. Though KOA can also occur during young adulthood, higher risk is observed among people over 45 years of age. An estimate predicts over 250 million patients are currently suffering from this disease globally [28]. Such a large number of KOA patients also have a deep economic cost on the overall society. As proposed by [28], KOA can be managed and treated much more effectively if detected early. As stated in [29], all non-pharmacological treatment measures of KOA, such as exercise routines and therapies, as well as pharmacological measures such as corticoid injections, have a higher success rate if implemented at early stages. Therefore, there is a huge requirement for a simple and easy technique to identify this disease in its infancy.

Presently, knee osteoarthritis is diagnosed through physical examinations and techniques such as X-ray, Magnetic Resonance Imaging (MRI) scan, and arthroscopy reports, among others. As mentioned in [30], X-ray and other radiographic images are commonly used techniques to determine the class of knee osteoarthritis due to factors such as cost and availability. Therefore, plain knee radiographs are predominantly used to evaluate KOA, as they effectively reveal osteophyte formation, reduction in knee joint space width, subchondral geodes, and subchondral bone sclerosis. Key characteristics of KOA often include joint space narrowing and bone spurs. However, most of these techniques, including radiographic images, have a high degree of subjectivity due to the difference in experience of the clinician making the diagnosis. Other techniques also have further limitations, such as high cost and dependence on the chondral anatomical location, among others.

### 2.2. X-Ray Grading: Kellgren–Lawrence Scale

**Figure 3:**
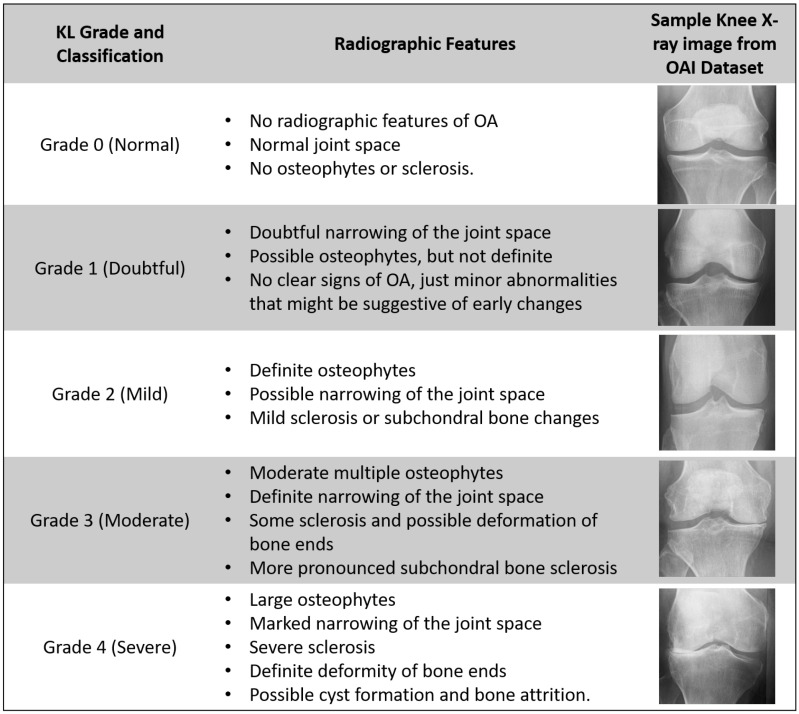
Kellgren–Lawrence grading scale for knee osteoarthritis with representative X-ray images.

The KL grading system serves as the standard for assessing OA. Recognized by the World Health Organization (WHO) as the standard in 1961, the KL grading system classifies OA severity into the following five stages: 0 (normal), 1 (doubtful), 2 (mild), 3 (moderate), and 4 (severe). KOA is a disease that is very hard to detect in the early stages when the distinction between grades 0 and 1 is very minimal. The classification according to the KL grading scale is dependent on the knee joint space width region area. Narrowing of knee joint space usually represents a higher degree and severity of KOA. As noted in [5], joint space narrowing results from ongoing damage to the articular cartilage, accompanied by the development of osteophytes, subchondral cysts, and subchondral sclerosis in the synovial joints, potentially leading to functional impairment. According to [31], Grade 0 represents the absence of Joint Space Narrowing (JSN) or reactive changes, Grade 1 suggests doubtful JSN and possible osteophytic lipping, while Grade 2 indicates clear osteophytes and potential JSN. Following this, Grade 3 signifies the presence of moderate osteophytes, definite JSN, some degree of sclerosis, and possible bone-end deformities, while Grade 4 denotes extensive osteophytes, pronounced JSN, severe sclerosis, and definitive bone-end deformities. Figure 3 displays the sample X-ray images with respective characteristics of different KL grades.

### 2.3. MRI-Based Grading Systems

While the X-ray images are widely used for KOA classification, they have limitations in assessing soft tissue structures and early osteoarthritic changes. MRI provides a more comprehensive evaluation of KOA by capturing cartilage integrity, bone marrow lesions, synovitis, and meniscal abnormalities. Several MRI-based grading systems have been developed to provide a quantitative assessment of KOA severity. The prominent MRI-based grading methods are Whole-Organ Magnetic Resonance Imaging Score (WORMS) [32], Boston-Leeds Osteoarthritis Knee Score (BLOKS) [33], and MRI Osteoarthritis Knee Score (MOAKS) [34].

1. WORMS: This system is one of the most widely used MRI-based grading systems for KOA categorization. It evaluates multiple joint structures, including cartilage morphology, bone marrow lesions, menisci, synovitis, and joint effusion. Each structure is graded separately, providing a comprehensive assessment of disease progression. WORMS is particularly useful in longitudinal studies to monitor KOA development over time.
2. BLOKS: BLOKS is another MRI-based grading system designed to assess KOA features related to disease progression. It focuses on specific biomarkers of joint degeneration, such as cartilage loss, bone marrow lesions, and synovitis/effusion. Compared to WORMS, BLOKS places greater emphasis on inflammation-related changes, making it useful for understanding the role of synovitis and effusion in KOA progression.
3. MOAKS: MOAKS is an advanced grading system that builds upon WORMS and BLOKS, integrating their strengths while addressing some of their limitations. It provides detailed scoring for cartilage damage, bone marrow lesions, osteophytes, meniscal integrity, and synovitis. MOAKS offers improved inter-reader reliability and is widely used in clinical research to quantify structural changes in KOA.

The KL grading system that uses X-rays mainly looks at bone spurs and joint space narrowing, but it does not detect early KOA or soft tissue changes. MRI-based systems such as WORMS, BLOKS, and MOAKS give a more detailed view of the knee by showing cartilage damage, bone marrow changes, meniscus problems, and inflammation. These systems are useful for tracking disease progression and severity in research and clinical studies. However, MRI is more expensive, takes longer, and requires special equipment compared to X-rays. Combining MRI-based grading with KL grading gives a clearer understanding of KOA and helps with better diagnosis, monitoring, and treatment planning.

## 3. Literature Review Methodology

The first step of every research project is to explore related studies and set eligibility criteria to specify which studies to include in the review. The literature review in this paper is carried out as explained in the following subsections.

### 3.1. Sources of Literature

Credible and trustworthy sources have been consulted to ensure the study’s integrity, with Google Scholar being extensively utilized for initial investigations. Research articles from reputable publishers such as IEEE, Elsevier, Wiley, ACM, ArXiv, Springer, etc. have been examined and incorporated into the research. Table 2 shows the publisher-wise distribution of research studies taken into consideration after various rounds of selection.

### 3.2. Inclusion and Exclusion Criteria

Precise delineation of inclusion criteria is essential for selecting relevant research articles, as it defines the scope and limitations of the review, aiding reader comprehension [35]. The selection criteria for shortlisting research papers for this research are as listed below:

1. Study that preferably proposes a model developed using publicly available datasets such as Osteoarthritis Initiative (OAI) and Multicenter Osteoarthritis Study (MOST), etc.
2. The research paper should be published in well-reputed journals.
3. The paper included should be a recent study to keep this research up-to-date.
4. Study proposing new methodologies to automate classification or reviewing existing literature or surveys on OA and KOA to keep this research as relevant as possible.
5. Study using preprocessed enhanced images.
6. Study that uses DL-based classification algorithms mostly using CNN-based architecture.
7. The study thoroughly details their research and includes evaluation metrics like accuracy, precision, and recall for the proposed model.

Exclusion: We have excluded the articles that satisfy the following criteria:

1. Articles that discuss only traditional image processing and ML techniques for KOA classification.
2. Articles that use or propose DL architectures other than CNN-based ones, such as autoencoders, transformers, etc.
3. KOA studies focusing on KOA progression based on the patient’s history.
4. Studies using other grading methods except KL grading for X-ray images.
5. Studies that use data modalities other than X-ray and MRI.

**Figure 4:**
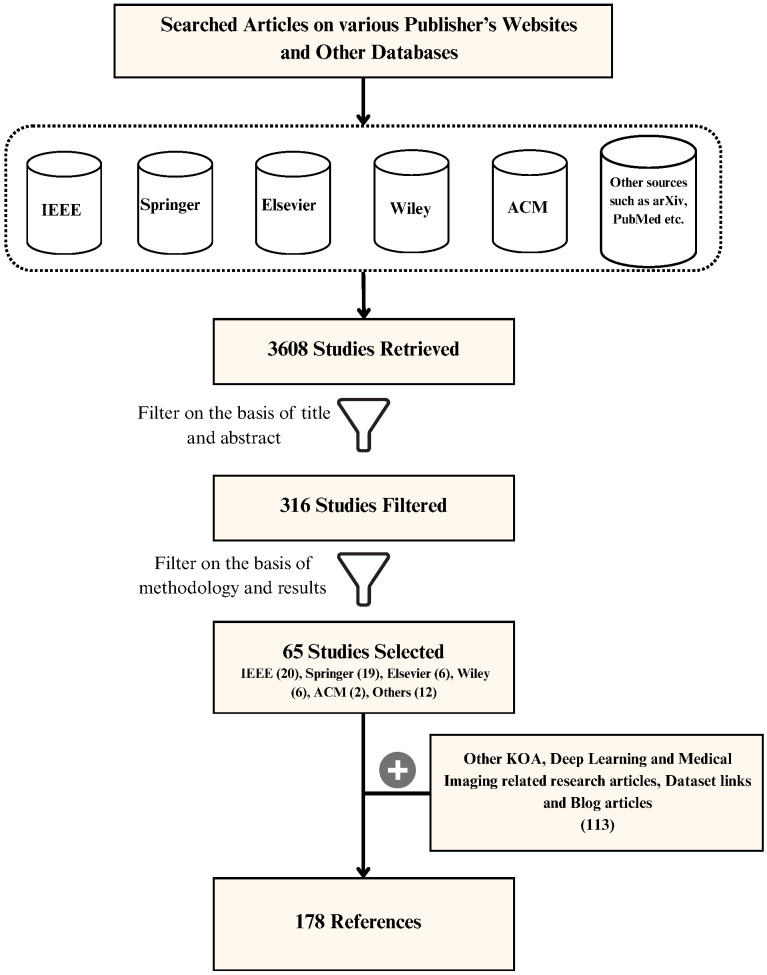
Research articles included after systematic screening.

**Figure 5:**
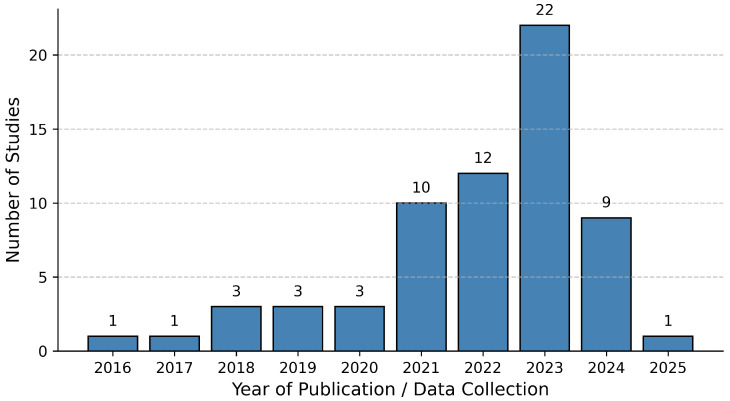
Year-wise distribution of studies included in the review (search window: 2016–2025).

The number of research studies and articles filtered at different stages while following the mentioned steps is shown in Figure 4. Figure 5 shows the year-wise distribution of KOA classification, DL, medical imaging, and other related research articles included in this paper.

### 3.3. Study Selection Process

The overall process for selecting, filtering, and including research studies in this review and comparison with other studies involved multiple steps:

1. Searching for papers using keywords such as “KOA”, “OA”, “KL Grade”, and “DL in Healthcare”, etc.
2. Optimizing the search to include only the studies published by reputed journals.
3. Going through the title and abstract of the study to decide its usefulness for the review.
4. Analyzing all the findings and listing the ones that can be used in the review study.
5. Noting the data sources and preprocessing techniques for the KOA classification studies.
6. Listing all the architectures proposed, fine-tuning methods used, and results obtained by the studies.
7. Mentioning the findings of the research in the appropriate section of this review and citing it.
8. Comparing the performance measures of solutions proposed by different studies using some common evaluation metrics.
9. Representing this survey visually in the form of appropriate figures, tables, graphs, and charts.

## 4. Deep Learning in Healthcare

Traditional diagnosis methods suffer from limitations such as subjectivity in the diagnosis, reach of the services to a larger population, affordability of diagnostic solutions, etc. These limitations present the need for automated, affordable, and efficient approaches with consistent results. The growing application of artificial intelligence (AI) through advanced ML and DL algorithms is helping experts by either fully or partially automating the diagnostic process. DL methods focus on constructing layered models that allow computers to autonomously perform tasks such as classification and object detection. As mentioned in [9], DL refers to ML programs developed based on “neural networks”, which are inspired by the neural network structure of the human brain and can adapt themselves through repetitive training to recognize patterns. DL is used for supervised learning, including image classification, image generation, object detection, and image segmentation. It is also used in the fields of unsupervised as well as reinforcement learning. In the vast field of image classification, many popular pre-trained models exist, such as VGGNet [36], ResNet [37], YOLO [38], DenseNet [39], MobileNet [40], and EfficientNet [41], etc.

According to [8], these complex DL models have shown excellent similarity with human experts in KOA detection and classification. Transfer Learning (TL) is used by these models, where knowledge from previously trained models is leveraged to address new tasks with minimal additional training or fine-tuning. Transfer learning is a two-phase process for training DL models, involving an initial pre-training phase followed by a fine-tuning phase where the model is adapted to the target task [42]. Thus, the classification of knee X-ray radiographs according to the KL grading scale can also be implemented by utilizing DL models effectively. After training the model, it can be fed new data, and predictions will be generated representing the severity of KOA. As researched upon in [43], DL techniques have been applied to numerous healthcare problems, including medical imaging, computer-aided detection/diagnosis, disease prediction, image segmentation, image generation, etc. Image classification is helpful in the identification of skin diseases in dermatology, eye disease recognition in ophthalmology, and classification of pathological images for various cancers such as breast cancer and brain cancer [44].

### Architectures and Applications

DL algorithms use complex multi-layer dense networks of neurons to learn the hidden patterns in the training data. As discussed in detail in [45], the CNN architectures first implement segmentation to group similar portions of an image into a single class and assign them labels. The next step in model building is the detection of features that indicate the presence and severity of KOA. Lastly, classification is done by grouping images having similar features and indicators into the same output classes. It is observed that the CNNs have superior performance with image, speech, and audio signal inputs.

CNNs consist of convolutional, pooling, and fully connected layers. The convolutional layer learns filter weights and biases via backpropagation and gradient descent to detect image features, generating multiple feature maps representing different characteristics of the input tensors [46]. Weight sharing reduces the number of parameters [47]. Common activation functions for classification include ReLU, Sigmoid, Tanh, Leaky ReLU, and SoftMax (Table 3). Consequently, CNNs are widely applied in image classification tasks, including medical imaging.

**Figure 6:**
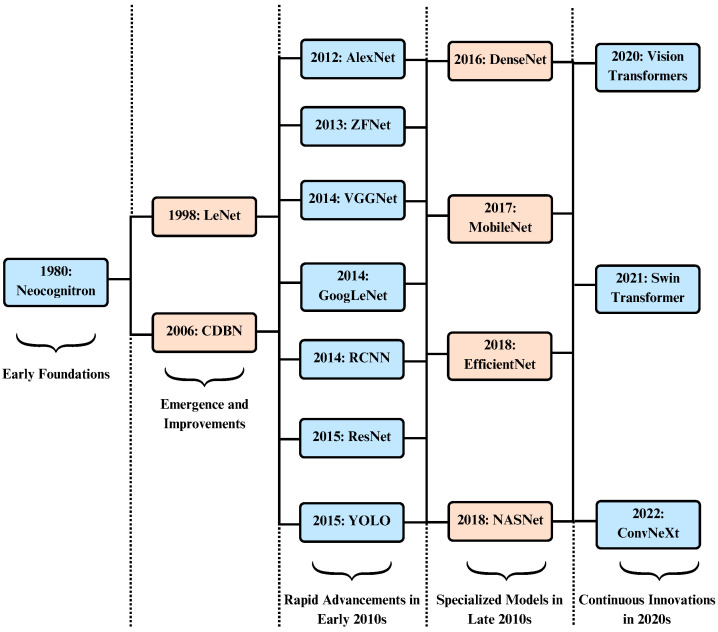
Chronological evolution of CNN architectures over the years.

Figure 6 illustrates the chronological development of CNN architectures over the years. Table 4 summarizes the key features and representative use cases of these models. Widely adopted pretrained networks include VGGNet, ResNet, Inception, YOLO, DenseNet, and EfficientNet [48].

**Figure 7:**
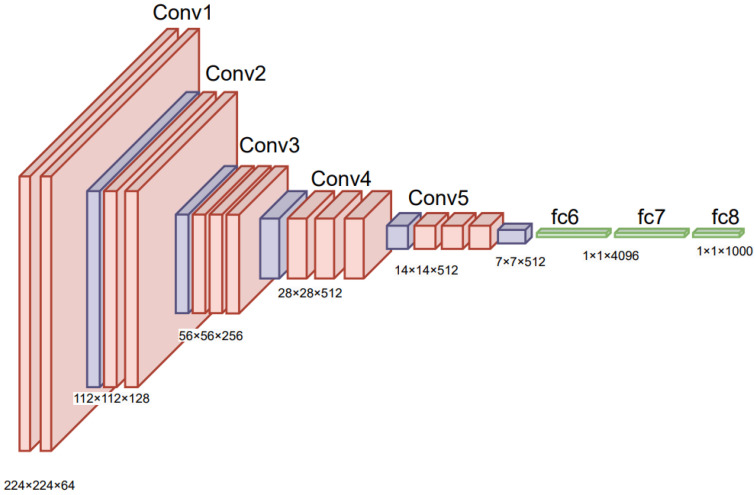
VGG16 convolutional neural network architecture [68].

The Visual Geometry Group (VGG) network architectures were developed by a team of researchers from the Department of Science and Engineering at Oxford University. The most widely used models released by this group are VGG16 and VGG19. VGG16 is a convolutional neural network with a structure of 16 layers, comprising 13 convolutional layers and 3 fully connected layers. The initial two convolutional layers utilize 64 feature kernels each [68]. The output is then fed into a max pooling layer with a stride of two. The third and fourth convolutional layers use 128 feature kernels each, followed by another max pooling layer with a stride of 2. The fifth, sixth, and seventh layers employ 256 feature maps, and eighth to thirteenth layers use 512 kernel filters. The final layers utilize max pooling with a stride of 1. The fourteenth and fifteenth layers consist of fully connected hidden layers with 4096 units each, concluding with a Softmax output layer with 1000 units. Figure 7 shows the layered architecture of VGG16.

ResNets are a type of deep neural network architecture that uses residual blocks that contain skip connections or shortcuts that are used to enable the model to learn residual functions. Several ResNet architectures have been proposed, such as ResNet-18 and ResNet-34. ResNet-50, ResNet-101, and ResNet-152. The most popular is the ResNet-50 model that uses a bottleneck block, which reduces computational complexity while increasing the depth of the network. It has a total of 50 layers, and the skip connections enable faster convergence during training by mitigating the vanishing gradient problem. Residual learning is applied to every few stacked layers within these architectures. The residual mapping operation is performed using shortcut connections that are inserted to turn it into a residual version [37].

The researchers in [69] proposed the Inception architecture with Pure Inception blocks and Residual Inception blocks. The inception modules provide the capability to learn diverse features at various levels of abstraction using parallel convolutional filters. These multiple outputs are then concatenated along the depth dimension. As a result, the Inception architecture can incorporate both local and global information and can implement better discrimination of different patterns in the data. Inception takes into account the model of transformers, which are widely used in the domain of Natural Language Processing (NLP) [70].

DenseNets are created to overcome issues found in traditional deep neural networks, such as the vanishing gradient problem and challenges associated with training very deep networks. It was introduced by Huang et al. in 2017 [39]. DenseNets consist of Dense Blocks, which consist of a series of convolutional layers with a fixed number of filters. The Transition Block controls the growth of the number of feature maps and reduces spatial dimensions. Global average pooling calculates the mean value of each feature map, resulting in a single value per channel. DenseNets use Multiscale Image Pyramids for CNNs, data centering through simplified RGB mean subtraction, can handle multiple aspect ratios, and provide a considerable speedup in computation time [71].

YOLO’s core concept involves partitioning the input image into a grid and conducting object detection over the whole image in a single forward pass through the neural network. The YOLO architecture [38] processes the entire image as input, divides it into a grid, and assigns each grid cell the task of predicting bounding boxes and class probabilities. The YOLO architecture has better speed and the ability to detect multiple objects in real-time. The authors in [72] summarize the different versions of YOLO architecture developed throughout the recent years, and it is observed that the use of batch normalization and high-resolution classifiers enhances the performance measures of YOLO. The YOLO architecture has better speed and the ability to detect multiple objects in real-time. The research [72] summarizes the different versions of YOLO architecture developed throughout the recent years. The use of batch normalization and high-resolution classifiers enhances the performance measures of YOLO.

## 5. Datasets for KOA

The task of building and testing DL models for KOA detection and classification requires a substantial amount of input data for the model training and validation process. These data have been obtained in various forms of images, like X-rays and MRIs, from a diverse range of sources [12]. The data collection process is detailed in the subsequent sections, outlining the primary types/modalities and data sources employed by different studies.

### 5.1. Imaging Modalities

The data used for detection and classification of KOA and its severity can be in various image forms. These include X-rays, MRI images, ultrasound images, CT scan images, and thermal images among others.

**Figure 8:**
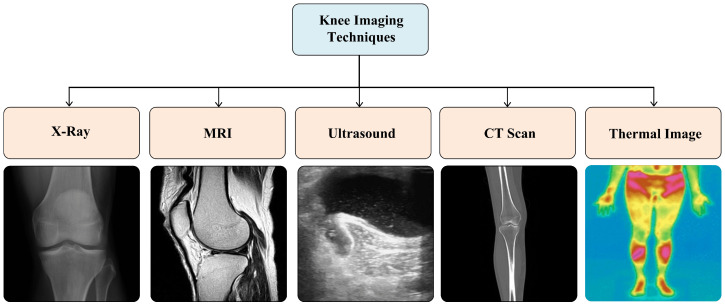
Representative knee images illustrating commonly used imaging modalities: X-ray [80], MRI [81], ultrasound [77], CT [78], and thermal imaging [79].

X-ray imaging is a crucial imaging technique in the medical domain that utilizes X-rays to create images of internal body structures such as bones and joints [73]. X-ray imaging is fast, efficient, cost-effective, and has high accuracy in detecting fractures, tumors, foreign objects, and abnormalities in various body systems [74]. In KOA assessment, X-ray images are mainly used to identify bony changes such as joint space narrowing and osteophyte formation; however, they have limited ability to detect early-stage disease and poor sensitivity for soft tissues such as cartilage, menisci, and ligaments. Conversely, MRI uses a strong magnetic field, radio waves, and computer technology to produce detailed images of organs, tissues, and other bodily structures. Unlike X-rays or CT scans, MRI does not involve ionizing radiation, making it a safer option for patients [75]. According to [76], MRI provides several benefits as a medical imaging modality, such as superior soft tissue contrast, multi-planar imaging, absence of ionizing radiation, high spatial resolution, functional imaging abilities, safety, versatility, and real-time imaging. Due to its superior soft tissue contrast, MRI is more suitable for early diagnosis of KOA and for accurately detecting cartilage degeneration, meniscal damage, and bone marrow lesions that are not visible in X-ray images. Additional methods include knee ultrasound [77], which employs high-frequency sound waves to generate real-time images of the knee joints and allows for immediate assessment of soft tissues like cartilage and menisci. The computed tomography (CT) scanning is another imaging technique used. A CT scan is a method that creates detailed cross-sectional slices of the knee joint [78]. Thermal images, or infrared thermography, is a recent imaging technique used in healthcare, which is a painless and non-invasive technique for the early detection of diseases. The resultant images display a color-coded distribution of the temperature of the examined area [79]. The knee image samples of each of these techniques are shown in Figure 8. Overall, X-rays and, to a lesser extent, MRIs are most commonly used for deep learning-based KOA severity classification, as they provide complementary information on bony and soft tissue changes.

### 5.2. X-Ray and MRI Dataset Sources

Various projects and initiatives were carried out across the world to collect and publish credible datasets of knee images. Some of the image datasets are publicly available and are widely used by researchers all over the world to develop automated KOA detection and classification models. Some of the major datasets are listed in the following subsections.

#### 5.2.1. OAI and MOST Datasets

Datasets for knee osteoarthritis cases have been collected from a wide range of sources across multiple studies. The most prominent sources are the OAI dataset [82] and the MOST [83] dataset. The OAI dataset consists of a large collection of knee X-ray radiographs and is publicly available. Different distributions of these datasets have been published by multiple researchers across the world. One of the most widely used distributions is the Chen distribution published in 2018 [84], consisting of 8260 unique images. Many other distributions have also been published, as highlighted in Table 5. It is analyzed that a large number of research studies have used these datasets to build and evaluate DL models for KOA classification based on KL grading [5,85,86,87,88,89,90,91,92,93,94,95,96,97]. The MOST dataset involves images collected from a longitudinal, prospective observational study of KOA in older Americans, either with existing OA or at higher risk of developing it. This dataset has been used in a number of KOA classification studies [94,96,97].

Along with X-ray images, the OAI and MOST datasets also contain a large number of knee MRI images. Along with training models to perform KOA classification using MRI images, some researchers have also used both X-ray and MRI images to predict KOA severity [98]. Authors in [81] have used MRI images from the OAI dataset for KOA classification; however, due to the 3D nature of the MRI scans, which are a sequence of 2D images, researchers [99] find it useful to use the MRI scans to analyze and predict the KOA progression instead of KOA classification. Similarly, the authors in [100] utilized these MRI scans to develop a DL-based explainer, which provides interpretability for KOA classification based on KL severity grading.

#### 5.2.2. Other Datasets

Other significant datasets used in KOA studies are as follows: MRNet [101], FastMRI [102], FASTMRI+ [103], and other datasets along with data collected from local hospitals. The MRNet dataset, released by Stanford ML group, contains 1370 MRI scans. These scans include 1106 abnormal scans capturing Anterior Cruciate Ligament (ACL) tears and meniscal tears prominently. The FastMRI dataset contains both MRI k-space and Digital Imaging and Communications in Medicine (DICOM) images obtained through MRI knee examinations [102]. The dataset contains 1594 k-space data images and 10,012 DICOM images. The FastMRI+ dataset [103] extends the FastMRI dataset by providing pathological annotations by clinical experts, which are critical for reconstruction frameworks. This dataset contains 16,154 bounding box annotations for different pathology categories originally released by thte FastMRI knee dataset. Many researchers have also sourced KOA X-ray samples from publicly available medical experts data [87,104] and data from local hospitals [87,105,106,107,108]. Some researchers [109] have also carried out community-based studies to obtain knee X-ray images. The number of publicly available datasets for MRI images [82,83] is less as compared to those of X-ray images. Hence, most of the studies using MRI as input data had to rely upon sourcing images from local hospitals [110] and community samples [111].

Table 5 and Table 6 present the details of the different X-ray and MRI datasets considered by some prominent studies for this research problem, respectively.

**Table 5:** Summary of X-ray datasets used in KOA studies, including dataset details, number of images, and image dimensions.

Reference	Year	Dataset Detail	No. of X-Ray Images	Image Dimension (Pixels)
**OAI Dataset along with its variants and MOST Dataset**				
Sohail et al. [112]	2025	OAI-modified by Chen [84]	8260	299 × 299
Ahmed et al. [85]	2024	OAI-obtained from Mendeley	8260	–
Malik et al. [86]	2024	OAI-obtained from Mendeley	5778	299 × 299
Touahema et al. [87]	2024	OAI (labeled by Boston University)—obtained from Mendeley	4446	224 × 224
Patil et al. [88]	2024	OAI	2250	384 × 384
Mohammed et al. [89]	2023	OAI obtained from Kaggle	9786	224 × 224
El-Ghany et al. [90]	2023	OAI assessed by Boston University X-ray reading center (BU)	4446	224 × 224
Guida et al. [98]	2023	OAI [Subset-1: both MRI and X-ray, Subset-2: Only X-ray]	Subset1: 1100 Subset2: 8821	MRI (160 × 160), X-ray (600 × 220)
Pi et al. [91]	2023	OAI-modified by Chen [84]	8260	224 × 224 (Model tested with different image sizes)
Pongsakonpruttikul et al. [5]	2022	OAI-modified by Chen [84]	1650	224 × 224
Wang et al. [92]	2021	OAI	4506	224 × 224
Yunus et al. [93]	2022	MOST	3795	224 × 224
Swiecick et al. [94]	2021	MOST	18,503	700 × 700
Norman et al. [95]	2019	OAI	39,593	500 × 500
Tiulpin et al. [96]	2018	MOST: for training, OAI: for validation and testing	18,376	224 × 224
Antony et al. [97]	2017	OAI & MOST	OAI: 4446 MOST: 2920	256 × 256
**Other datasets and dataset from local hospitals**				
Touahema et al. [87]	2024	Medical Expert Public Dataset—collected from various hospitals and diagnostic centers in India	1650	362 × 162
Touahema et al. [87]	2024	El Kelaa des Sraghna Provincial Hospital	30	–
Alshamrani et al. [104]	2023	Dataset obtained from Kaggle	3836	224 × 224
Hengaju et al. [105]	2022	Bhaktapur Hospital	350	256 × 256
Abdullah et al. [106]	2022	Radiological center (KGS scan center, Madurai)	3172	3000 × 1500
Sikkandar et al. [107]	2022	Durma and Tumair General Hospital, Riyadh	350	256 × 256
Olsson et al. [108]	2021	Danderyd University Hospital	6403	256 × 256
Shamir et al. [109]	2009	Baltimore Longitudinal Study of Aging (BLSA)	350	1000 × 945

**Table 6:** Summary of MRI datasets used in KOA studies, including dataset details, number of images, and image dimensions.

Reference	Year	Dataset Detail	No. of Knee MRI	Image Dimension (Pixels)
Guo et al. [113]	2024	OAI + FastMRI + SKI10 + private	700	–
Guida et al. [98]	2023	OAI [Subset-1: both MRI and X-ray, Subset-2: Only X-ray]	1100 (number of knees)	After crop: 160 × 160
Harman et al. [114]	2023	FastMRI+	663	–
Hung et al. [115]	2023	private (584) + MRNet (120)	704	512 × 512
Schiratti et al. [99]	2021	OAI[ 2D MRI images of type “COR IW TSE”	9280	–
Karim et al. [100]	2021	MOST [2406 patients with MRI data]	4678 MRI slices	Re-scaled to 360 × 360
Guida et al. [81]	2021	OAI [3D DESS MRI—a sequence of 160 2D images]	1100	384 × 384
Du et al. [116]	2018	OAI	4800	448 × 448
Kumar et al. [110]	2016	SRM Medical College Hospital and Research Center	15	256 × 256
Marques et al. [111]	2013	Community based, Non-treatment Study	268	170 × 170

### 5.3. Dataset Provenance, Label Reliability, and Data Hygiene

KOA studies use large public datasets such as OAI and MOST, which provide bilateral and longitudinal knee radiographs graded using the KL scale [94,112,117]. These datasets follow standardized imaging protocols and employ trained readers. However, KL grading is based on visual assessment and is known to show variability between readers, especially for neighboring grades such as KL 1-2 and KL 2-3. Several studies attempt to reduce this variability by using consensus grading, third-reader adjudication, or selecting the modal grade from multiple readings [92,95,109]. Despite these efforts, some degree of label noise remains unavoidable in X-ray-based KOA datasets.

Another important concern is subject-level dependency. Both OAI and MOST are longitudinal studies in which multiple images may exist for the same participant, including left and right knees and repeated follow-up visits [94,95,117]. If data splitting is performed at the image level instead of the patient level, images from the same individual may appear in both training and testing sets. This can lead to information leakage and overly optimistic performance results. The risk is higher when the left and right knees are treated as independent samples without enforcing subject-wise separation [95,112].

Longitudinal duplication is another source of bias, as radiographs from different time points of the same knee remain highly correlated [94,117]. Some studies explicitly control this issue by restricting analysis to baseline images, using a single knee per subject, or applying subject-wise data partitioning [116,117]. Similar challenges exist in MRI-based studies derived from OAI, where repeated scans and expert annotations may also introduce correlated samples despite richer structural information [98,116]. Table 5 and Table 6 summarize the X-ray and MRI datasets used in KOA studies.

## 6. Data Preparation and Model Development

**Figure 9:**
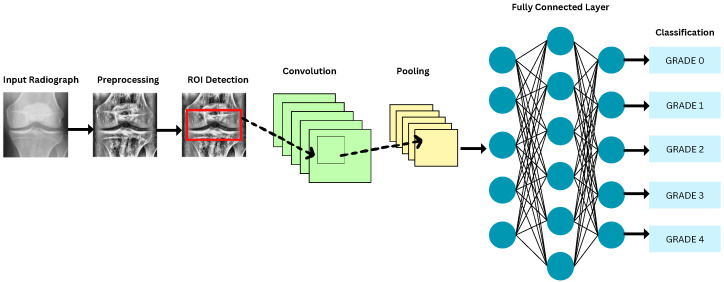
General workflow of deep learning-based KOA detection and classification using the KL grading system, including data preprocessing, Region of Interest (ROI) detection, model development, and evaluation steps.

Recent research articles often introduce different DL architectures for detecting and classifying KOA using the KL grading scale, and then evaluate these architectures’ performance using metrics such as accuracy, precision, recall, and F1-score. The input data are generally first augmented to provide more exposure while training the DL-based model in a balanced proportion of all the classes. The data are subsequently processed to improve image quality and extract more valuable information, leading to better prediction outcomes. The refined data are input into DL models for detecting and classifying KOA according to the KL grading system. The general steps in most of the research studies are shown in Figure 9. The following subsections describe various building blocks of the automatic KOA detection and classification system.

### 6.1. Data Augmentation

The majority of the datasets, irrespective of their sources, are highly imbalanced in terms of KL Grade classes. As a result, the models trained on such datasets could generate predictions biased towards a particular class and affect the credibility of the results. Oversampling the minority classes is required to make the datasets more balanced. This involves augmenting the dataset with new images generated by randomly varying the features of existing images. For this, variations to the properties of existing images are applied, and new images are created. These included rotating images from −3 to 3 degrees, varying at every 45 degrees, and adjusting brightness and contrast by multiplying all pixel red, green, and blue values by steps ranging from 0.6 to 1.0 [5]. Some studies have also applied color jittering techniques to randomly alter the brightness, contrast, and saturation of the images in the dataset.

One of the popular approaches to generating augmented data is the TensorFlow ImageDataGenerator library. This provides inbuilt functions to augment given data by adjusting parameters such as brightness, contrast, rotation, width, height, shift, flip, and zoom, etc., of the original set of images. Another method widely used is to upscale the number of minority class images by varying parameters using a Python 3.12 program. It is observed from the research studies that a balanced dataset is more useful for training DL models with better accuracy scores and better prediction rates.

### 6.2. Preprocessing Methods

The images obtained from knee X-ray radiographs may have a lot of noise and distortions, class imbalance, and are not suitable for applying DL techniques directly. Hence, preprocessing techniques to obtain high-quality data are necessary for better performance of DL models. The most basic preprocessing step is to resize the images into sizes acceptable by pre-trained models. Authors in [89] discarded excess information in the image by cropping 60 pixels, which are cropped from both the top and bottom of each image.

**Figure 10:**
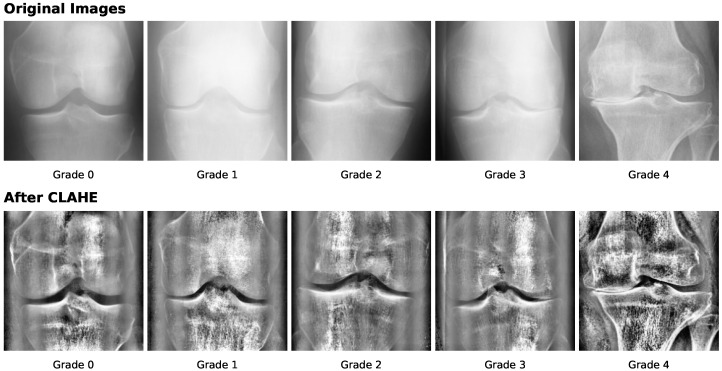
Illustration of the effects of applying CLAHE on sample knee X-ray images across different KL grades.

Most studies use contrast enhancement as a primary preprocessing step to improve image quality by modifying intensity distributions. Histogram Equalization (HE) enhances image contrast by redistributing pixel intensities to achieve a roughly uniform histogram [118]. Brightness-preserving HE maintains both bright and dark areas while improving overall contrast [119]. Common image enhancement techniques, including HE, contrast stretching, sharpening filters, and Gaussian smoothing, help detect subtle features like joint space narrowing or osteophytes. Key parameters include grid size (number of image tiles) and clip limit (threshold to prevent over-amplification). Adaptive Histogram Equalization (AHE) applies equalization locally on tiles (commonly 8 × 8 with clip limit 2.0) to adjust local contrast, improving overall image quality. Contrast Limited Adaptive Histogram Equalization (CLAHE) [120,121] further refines this by limiting histogram heights per tile, preserving fine details while enhancing contrast. Focusing on the main region of interest often improves results by removing irrelevant image information. The effect of CLAHE on sample knee X-ray images of each KL grade is illustrated in Figure 10.

In [105], the authors removed noise from the image by preserving bone edges and using an adaptive median filter. Image enhancement is done to improve the perception of information in the image. It involves improving the quality in terms of contrast, sharpness, masking, etc. The study [104] uses denoising techniques to remove noise from the X-ray images. This involves applying a 2D median filter with a kernel size of 5 × 5. Authors in [90] used artifact removal, resizing, contrast handling, and normalization to obtain more useful images. In [122], authors used contrast stretching, histogram equalization and Gaussian filters on MRI images to improve the quality of the images. In [117], the data with missing labels was removed from both OAI and MOST datasets. In [92], the authors converted X-ray images into standard 8-bit grayscale images using the Pydicom toolkit to make data more consistent. The studies [93,116] used Principal Component Analysis (PCA) for the selection of the most optimal features. In [111], partial least square regression was used for feature selection and extraction from MRI images.

In [110], the input MRI images are initially resized to 512 × 512 pixels and then converted to grayscale, followed by contrast enhancement and histogram equalization. A thresholding operation is subsequently applied to remove pixels with low intensity values, and background noise is removed. The authors of [123] use global non-rigid registration followed by four local registrations for image preprocessing before segmentation of the desired region of interest. Overall, nearly all studies apply preprocessing techniques to their respective datasets prior to implementing DL models. These techniques are summarized in Table 7.

### 6.3. Segmentation Approaches

**Figure 11:**
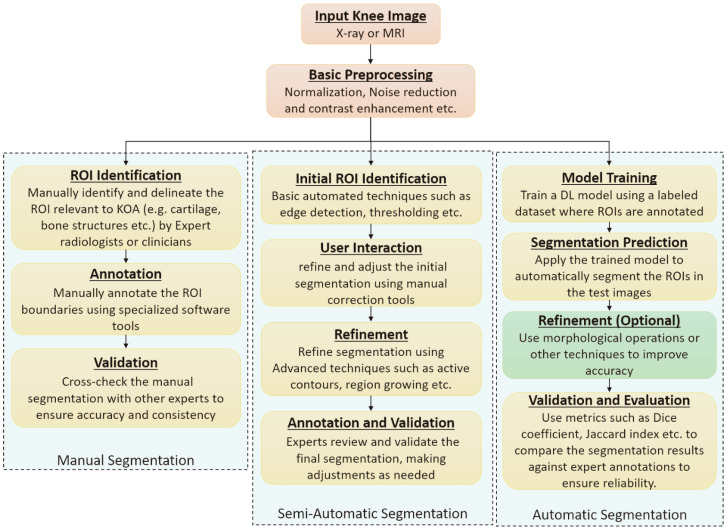
Overview of manual, semi-automatic, and automatic segmentation techniques for knee radiographs.

Proper placement of the ROI is essential for analyzing bone texture in the assessment of OA. Proper localization enhances prediction accuracy. Several studies emphasize the impact of ROI size, shape, and position on OA prediction in knee texture-based methods [128]. ROI detection is the process of localizing and identifying specific regions of importance within an image or dataset for further analysis. ROI detection is carried out by placing bounding boxes in the images around the region to locate the joint space width in radiographs and articular cartilage in MRIs. It is the first step in KOA classification, as stated in [129]. The VGG Image Annotator (VIA) tool was used in [127] to enclose each knee joint with bounding boxes in the images considered. Bounding boxes limit the image to its most informative part and make it easier for the DL models to extract useful features for classification. The authors in [107] performed segmentation using local center of mass algorithms to extract ROI. This results in dividing the image into more meaningful and homogeneous regions for better separation and analysis of components. In [117], to precisely capture ROI, the BoneFinder tool uses a Random Forest regression voting method to identify knee joint landmarks. In [105], active contour segmentation method was used for finding regions of interest. From the literature, it is analyzed that three types of segmentation approaches are used to identify, annotate, and validate the ROI in knee radiographs, namely, manual segmentation, semi-automatic segmentation, and automatic segmentation approaches. The process followed by each approach is broadly shown in Figure 11.

Beyond accurate localization, the choice of ROI strategy directly influences downstream KOA classification performance. Manual ROI selection can provide precise joint localization but introduces reader-dependent variability and limited scalability, which affects reproducibility [130,131]. Semi-automatic methods reduce annotation effort while preserving anatomical consistency; however, their performance depends on initialization quality and parameter tuning [132,133]. Fully automatic approaches enable large-scale and consistent ROI placement but are sensitive to mislocalization and background inclusion. Landmark-based automatic methods, such as BoneFinder, provide anatomically aligned ROIs and more stable feature extraction, which has been shown to improve classification robustness in knee radiograph analysis [117]. Detector-based methods, including YOLO and Faster R-CNN, allow end-to-end ROI detection and classification, and have reported improved performance when trained on well-localized ROIs [5,106]. However, errors in bounding box placement can propagate to the classification stage and degrade performance, especially in advanced OA cases [128].

In MRI scans, accurate segmentation of cartilage is crucial for obtaining precise quantitative measures, including volume, thickness, and surface area, which are vital for detecting structural changes. Despite the challenge posed by cartilage morphology and MRI acquisition, robust segmentation is essential for reliable diagnostic and therapeutic decision-making in knee joint pathology [134]. Manual segmentation is the standard practice in knee cartilage segmentation. However, the correctness check of the segmented area is performed by the expert radiologists, and, therefore, it may introduce human bias in the accuracy of the segmented points. Table 8 summarizes various manual segmentation techniques along with the segmented knee area mentioned in the literature.

Computational methods for segmenting articular cartilage (AC) from MRI images employ advanced image-processing and pattern recognition techniques to extract relevant features, minimizing human intervention [136]. Segmentation approaches are generally categorized into semi-automatic and fully automatic models [133]. Accurate segmentation improves model interpretation and enables focused analysis. The semi-automatic segmentation methods used in KOA studies are summarized in Table 9.

Table 10 presents the fully automatic segmentation methods. Recent studies increasingly leverage DL models for segmentation, with UNet being one of the most widely adopted architectures.

Overall, segmentation should be viewed not only as a preprocessing step but also as a key factor influencing feature quality, classification robustness, and reported performance in KOA studies.

### 6.4. DL Models for KOA Classification

After preprocessing, model selection is a critical step in KOA severity classification. Most studies rely on pre-trained DL architectures, often adapted for knee joint analysis. DenseNet variants are popular due to efficient feature propagation. For instance, Ref. [90] employed DenseNet-169, achieving high accuracy (96%) on the OAI dataset, while DenseNet-121 was applied in [127] after ROI segmentation using Mask R-CNN and VGG Image Annotator, yielding moderate performance (73% precision, recall, F1-score). DenseNet-201 combined with EfficientNet-B0 for feature extraction, feature fusion, and dimensionality reduction via SVM and neural networks achieved 90% accuracy [119]. DenseNets capture fine-grained features effectively but require careful preprocessing to avoid noise amplification.

ResNet architectures, particularly ResNet101 and ResNet50, are widely used for classification. Reference [89] found ResNet101 most effective among six CNN models, achieving 69% accuracy. Two-step pipelines integrating ROI detection and classification, such as YOLO for ROI followed by ResNet50 for classification [92], achieve ROI detection confidence of 85% and classification accuracy of 69%. Modified YOLOv3 Tiny models also demonstrated strong performance for both KOA detection and severity classification (85–87%) [5]. Faster R-CNN generally provides more precise ROI localization than YOLO, with minimal joint space width detection achieving 99.6% accuracy [106].

VGG and AlexNet remain widely applied due to simplicity and ease of transfer learning. Fine-tuned VGG19 achieved 70% accuracy [126], whereas VGG16 reached 92% after extensive preprocessing, including denoising, contrast enhancement, and feature extraction [104]. Modified AlexNet with transfer learning also delivered high performance (99% accuracy) [106]. However, these architectures may underperform on subtle structural variations compared to deeper networks.

Advanced techniques include Siamese networks for medial-lateral knee similarity learning (62% accuracy) [124], hyper-parameter optimized DCNNs for joint space narrowing and osteophyte staging (77% accuracy) [143], and multimodal fusion models combining X-ray and MRI images (76% accuracy) [98]. Three-dimensional CNNs applied to MRI, such as 3D DenseNet, provide superior spatial feature extraction, achieving up to 96% accuracy for ACL lesion detection [144], while semantic segmentation CNNs with 3D deformable modeling also improve performance [145].

Classical ML remains relevant with feature-engineered approaches. SVM with PCA on MRI [116], Chan-Vese edge detection with SVM [122] (95%), GLCM features with SVM [110] (87%), HOG with multiclass SVM [27] (95%), and Fischer LDA [111] (0.92 AUC) demonstrate that well-engineered features can provide competitive results, though generally underperform compared to deep networks. Ensemble methods combining SVM, Random Forest, and Gradient Boosting also improve multiclass classification (87%) [30].

Overall, DL models dominate KOA severity classification, with DenseNet, ResNet, and VGG variants most frequently applied. Table 11 summarizes different ML and DL architectures used in reviewed articles. The popularly used metrics for evaluating the performance of a classification model are accuracy and F1-score. Other metrics include recall, precision, and specificity. For segmentation and ROI localization, YOLO or Faster R-CNN significantly improves performance, while multimodal or ensemble strategies represent the latest evolution in methodology. The comparison of the performance of different segmentation models is typically performed using the dice score, which computes the percentage of overlapping regions between the original and predicted segmentation masks. Table 12 and Table 13 compare the performance of different ML and DL based classifiers for multiclass and binary classification for KOA severity, illustrating the progression from classical ML and simple CNNs to sophisticated deep networks and multimodal frameworks.

KOA studies show that the selection of the DL model for KOA analysis depends on various factors such as the complexity of the knee structures, the type of imaging data used, and the required computational efficiency. DenseNet and EfficientNet perform well for detailed KL grading because they preserve and reuse features effectively, although they can be memory-intensive and sensitive to noisy preprocessing. ResNet provides a good balance between model depth and computational cost, making it a reliable general-purpose choice; however, it may miss very subtle structural variations in low-contrast images. Detection models such as YOLO and Faster R-CNN improve accuracy by precisely identifying the knee joint region before classification, but require additional training time and high-quality annotations. Simpler CNNs such as VGG and AlexNet remain useful for smaller datasets or as strong baseline models, but their limited depth reduces their ability to capture fine-grained KOA features. For MRI-based studies, 3D CNNs are preferred because they can capture volumetric information needed to assess cartilage and meniscal conditions, though they require large datasets and high computational resources. In general, each model has its own strengths and limitations, making the model choice dependent on the specific task and dataset characteristics.

**Table 11:** Summary of prominent machine learning and deep learning models employed for KOA severity classification.

Category	Architecture	References
Deep Learning	Residual Networks (ResNets)	[89,91,92,104,105,106,108,117,124,146,147,148,149,150,151,152,153]
	DenseNets	[80,89,90,91,95,100,119,123,127,152,154]
	Visual Geometry Group (VGG)	[89,94,100,104,105,152,155,156,157]
	You Only Look Once (YOLO)	[5,92,93,158]
	EfficientNet	[91,159,160]
	Region based CNN (R-CNN)	[94,106,127]
	MobileNet	[89,153,161,162]
	AlexNet	[106,163]
	Darknet	[164]
	Inception	[89,112,153]
	ShuffleNet	[91]
	NASNet	[165]
	HRNet	[166]
	LENET	[167]
	Deep Siamese Network	[124]
	UNet	[95]
	CaffeNet	[157]
Machine Learning	Support Vector Machines	[27,110,116,119,122,150,164,168,169,170]
	k-Nearest Neighbours	[93,109,169,171,172]
	Random Forest Classifier	[169,173,174]
	Naive Bayes Classifier	[174]
Hybrid Models	CNN with SVM, RF, and Gradient Boosting	[30]

**Table 12:** Performance Comparison of existing multiclass classifiers for KOA classification.

Reference	Year	Dataset	Test Set Size	ROI Method	Imbalance Handling	Validation	Key Performance
Sohail et al. [112]	2025	OAI	826	NR	Data Augmentation	internal	Acc: 92.25, F1: 92.30, K: 90.69
Ahmed et al. [85]	2024	OAI	1656	NR	NR	internal	Acc: 56.28, F1: 63
Touahema et al. [87]	2024	OAI	1000	NR	Data Augmentation	Internal	Acc: 97.20, F1: 97
Malik et al. [86]	2024	OAI	488	NR	Data Augmentation	internal	Acc: 89.89, F1: 78.25
Patil et al. [88]	2024	OAI	125	DFCN	NR	internal	Acc: 94
Mohammed et al. [89]	2023	OAI	1656	NR	None	internal	Acc: 67, F1: 67
El-Ghany et al. [90]	2023	OAI	1778	GradCAM	NR	internal	Acc: 95.93, F1: 87.08
Guida et al. [98]	2023	OAI	1755	NR	undersampling	internal	Acc: 76
Alshamran et al. [104]	2023	Kaggle	845	NR	stratified sampling	internal	Acc: 92.17, F1: 92
Tariq et al. [152]	2023	OAI	1656	NR	None	internal	Acc: 98, F1: 97, K: 99
Haseeb et al. [119]	2023	Kaggle	2348	NR	NR	internal	Acc: 90.1, F1: 88
Aladhadh et al. [154]	2023	Mendeley VI, OAI	2500	CenterNet	NR	external	Acc: 99.14, F1: 99.44, Dice Score: 99.24 ± 0.03
Kiruthika et al. [125]	2022	OAI, MOST	3500	FCN	NR	internal	Acc: 98.75, F1: 99.3
Pongsakonpruttikul et al. [5]	2022	OAI	150	Manual	undersampling	internal	Acc: 86.7, F1: 61.1
Abdullah et al. [106]	2022	private	634	RPN (Region Proposal Network)	NR	internal	Acc: 98.90, Dice Score: 98.90
Yunus et al. [93]	2022	Mendeley	1656	YOLOv2-ONNX	NR	internal	Acc: 90.6, F1: 88.0
Cueva et al. [124]	2022	OAI, private	225	NR	oversampling	external	Acc: 61.71
Sikkandar et al. [107]	2022	Private	70	Local Center of Mass (LCM)	NR	internal	Acc: 72.01, K: 86
Hengaju et al. [105]	2022	Private	140	Active Contour	NR	internal	Acc: 59
Kondal et al. [127]	2022	OAI, private	1175	Mask RCNN	NR	external	F1: 73
Swiecicki et al. [94]	2021	MOST	3359	RPN	NR	internal	Acc: 71.90, K: 75.9
Wang et al. [92]	2021	OAI	1660	YOLO	NR	internal	Acc: 69.18
Tiulpin et al. [117]	2020	OAI, MOST	11,743	Random Forest Regression Voting	NR	external	Acc: 67, K: 82
Norman et al. [95]	2019	OAI	5941	U-Net	NR	internal	Acc: 78.36
Pedoia et al. [123]	2019	OAI	657	Voxel Based Relaxometry	NR	internal	R: 76.99, Ssy: 77.94
Du at al. [116]	2018	OAI	100	NR	NR	10-fold CV	Acc: 70
Kumar et al. [110]	2016	Private	15	Pixel-based segmentation	NR	internal	Acc: 86.67

**Table 13:** Performance Comparison of existing binary classifiers for KOA classification based on KL grading.

Reference	Year	Dataset	Test Set Size	ROI Method	Imbalance Handling	Validation	Key Performance
Mohammed et al. [89]	2023	OAI	1656	NR	None	internal	Acc: 83, F1: 83
El-Ghany et al. [90]	2023	OAI	1778	GradCAM	NR	internal	Acc: 93.78, F1: 89.27
Pongsakonpruttikul et al. [5]	2022	OAI	100	Manual	undersampling	internal	Acc: 85, F1: 85

## 7. Discussion and Future Research Directions

This paper exhaustively analyzes various DL methods proposed by multiple research studies published across the world. The datasets used, preprocessing techniques, and model architectures have been thoroughly studied to figure out the best possible combinations to classify KOA according to the KL grading scale with the highest performance measures. The major shortcomings in present-day studies are listed below.

- Lack of availability of a balanced dataset to train the models makes them perform poorly for new and unseen data of the minority class.
- In an unbalanced dataset, traditional evaluation metrics such as accuracy become misleading as high accuracy can be achieved by simply predicting the majority class all the time, while still performing poorly on the minority class.
- The quality of the input images for model training requires multiple levels of preprocessing techniques to make them suitable for model training.
- In most of the available datasets, many images get discarded due to poor resolution or absence of ROI, which further depicts the problem of class imbalance.
- Requirement of a huge amount of computing resources to train such a large number of images.
- The labeling of the data points is done by radiologists, which introduces subjectivity in the overall process. The same knee X-ray image can be identified as belonging to separate KL grades by different radiologists. This makes the dataset available for training ambiguous and generates further inconsistency in predicting the actual severity of KOA.
- Potential data leakage can occur when images from the same patient, such as left and right knees or longitudinal scans, appear in both training and testing sets, leading to inflated performance estimates and reduced model generalizability.

After reviewing the utilization of various DL techniques by different authors for KOA classification based on severity grading, several research gaps have been identified. These gaps highlight opportunities for future research in this domain and also provide probable solutions for the limitations of existing approaches mentioned above.

- **Handling Class Imbalance and Performance Evaluation:** Class imbalance can reduce the performance of DL models if not properly addressed. Techniques such as over-sampling, under-sampling, and synthetic data generation can help balance the classes, and creating new datasets with more representative samples or combining data from multiple repositories can further improve model accuracy [175]. In addition, accuracy alone may be misleading for imbalanced datasets, so metrics like sensitivity, specificity, and F1-score should be used to evaluate and compare the performance of models, specifically for healthcare applications [176].
- **Enhancements in DL models:** Some of the studies [177] suggested that model computations can be optimized by changing the shape of the convolutional kernel and using texture memory. Other approaches can be explored to reduce the model computations. Collecting large amounts of malignant data for model training, using effective preprocessing techniques for the best feature extraction, and gathering information analysis about the knee can also further improve model performance.
- **Model Complexity:** The selection of more complex and accurate models that can deduce a better correlation between the pixel values in the preprocessed X-ray images and KOA severity according to the KL grading scale can improve the overall performance of DL-based models. With rapid improvements in the field of AI and ML and their applications, better and more accurate architectures are being proposed every year [178]. Therefore, newer architectures can be used to identify features in knee X-rays and classify them according to KOA severity.
- **Other efficient DL architectures:** The usage of Recurrent Neural Networks, Transformers, Reinforcement Learning, and Generative Adversarial Networks can also be explored for KOA detection and classification.
- **Multimodal Large Models:** Multimodal large models that combine knee images with clinical, demographic, or textual data can capture complex relationships between different data types. These models have shown strong performance in medical image analysis [179,180] and can help improve KOA classification accuracy and provide better interpretability.
- **Data Hygiene and Label Reliability:** Deep learning models for KOA classification strongly depend on the quality of training data and label consistency. Commonly used public datasets such as OAI and MOST rely on expert-assigned KL grades, which are subjective and show variability across readers, especially for borderline grades. This introduces unavoidable label noise. In addition, these datasets are bilateral and longitudinal, meaning that images from the same patient (left and right knees or follow-up visits) may appear multiple times. If data splitting is done at the image level instead of the patient level, data leakage can occur and lead to overestimated model performance. Therefore, future studies should apply patient-wise data splitting and clearly report dataset handling procedures. At present, KOA models are better suited for clinical support tasks such as triage and quality assurance rather than independent diagnosis.
- **Regulatory and Clinical Validation:** In addition to technical accuracy, KOA models require thorough clinical validation before deployment. This includes evaluation using standardized protocols, external testing on independent datasets, and clear reporting of dataset sources and validation strategies. Adherence to regulatory guidelines is necessary to ensure model safety, reliability, and clinical usefulness.

Overall, it is observed that there is a lot of scope for improving the overall accuracy of DL-based models on any kind of KOA X-ray and MRI data.

## 8. Conclusions

This review presents a comprehensive analysis of the recent progress and provides insights into the future directions for automated detection of KOA. It highlights how DL techniques can assist medical professionals, such as radiologists, in accurately detecting and classifying KOA. Numerous relevant studies were examined to explore the available datasets, preprocessing strategies, segmentation methods, and the variety of models employed for classifying KOA according to the Kellgren-Lawrence grading scale using X-ray and MRI images. Many of the studies reviewed present accuracy and F1 score values greater than 90%, though most approaches use an internal split to validate the results of the trained model. Using limited data from a single source undermines the reliability of the results and proves inadequate to claim generalizability. On the contrary, recent research focuses on developing multiclass classifiers instead of binary classifiers and thus improves the decision-making of the expert and KOA management for the patient. The review also identifies common shortcomings in existing studies, including limited dataset sizes, inconsistent preprocessing, and suboptimal model training. Addressing these challenges may involve generating larger, high-quality datasets, applying more effective preprocessing, and improving model training strategies. Future research should focus on integrating 3D imaging and multimodal MRI–X-ray data to enhance feature extraction and improve KOA severity prediction. This review mainly covered CNN-based models, while other deep learning approaches, such as autoencoders and transformer-based models, are also being used and could be explored in future work. 

## Figures and Tables

**Table 2:** Publisher-wise distribution of research studies and their selection at different screening stages.

Publisher	Total Studies Found	Initial Selection	Final Selection
IEEE	282	143	20
Springer	1381	49	19
Elsevier	194	28	6
Wiley	1044	27	6
ACM	182	12	2
Others	525	57	12

**Table 3:** Common activation functions in CNNs for classification, where *x* denotes the input from the previous layer before applying the activation function.

Activation Function	Formula
SoftMax	$f\left(x_{i}\right)=\frac{e^{x_{i}}}{\sum_{j}e^{x_{j}}}$
Sigmoid	$f\left(x\right)=\frac{1}{1+e^{-x}}$
Tanh	f(x)=tanh(x)
ReLU	f(x)=max(0,x)
Leaky ReLU	f(x)=max(0.01x,x)

**Table 4:** Key features and representative use cases of prominent CNN architectures.

Year	Architecture	Key Features	Example Use Case
1998	LeNet [49]	The initial successful implementation of CNNs involved five alternating layers of convolution and pooling, utilizing tanh or sigmoid activation functions	Recognizing handwritten and machine-printed characters, Face Recognition [50]
2012	AlexNet [51]	Uses ReLU activation function, use of dropout layers, trained on GPUs	Large-scale image recognition, surface defect recognition [52]
2013	ZFNet [53]	Less number of filters with reduced stride, retains more pixel information, visualization of features	Classification of ImageNet data, object classification [54]
2014	VGGNet [36]	Deeper networks with smaller filters, same depth of convolutional layers, has multiple configurations	Image classification, object detection, medical imaging, surveillance [55]
2014	GoogLeNet [56]	Use of Inception Module, more efficient computation and deeper networks, multiple version of inception	Image segmentation, transfer learning, video analysis, medical imaging [57]
2014	R-CNN [58]	Segmentation into regions of interest, generation of fixed length feature vector, use of bounding boxes and coordinates	Object detection, visual search, document analysis, ocr, autonomous vehicles [59]
2015	ResNet [37]	Use of skip connections to train deeper networks, overcome vanishing gradient problem, global average pooling after residual blocks	Semantic segmentation, medical image analysis, transfer learning, facial recognition, edge computing [60]
2015	YOLO [38]	Single forward pass and detection, divides image into grid cells, uses bounding boxes for feature detection	Security and surveillance, object tracking, drone applications [61]
2016	DenseNet [39]	Use of dense blocks where each layer is connected to every other layer in feed-forward fashion, feature reuse	Fine-grained recognition, object recognition in unstructured environments [50]
2017	MobileNet [40]	For mobile and embedded vision applications, Use depth-wise separable convolutions, reduced model size and complexity	Mobile and embedded vision applications, real-time object detection, inspection and defect identification [62]
2018	EfficientNet [41]	Uses compound scaling method, efficient architectural design with MBConv blocks, SE optimization, and the use of Swish activation function	Image classification, object detection and localization, and semantic segmentation [63]
2018	NASNet [64]	Use of neural architecture search and reinforcement learning, facilitates transferability and scalability	Medical imaging, autonomous vehicles, and industrial quality control [65]
2022	ConvNeXt [66]	It integrates vision transformers, layer normalization, and the Gaussian Error Linear Unit (GELU) activation function	Pedestrian and traffic sign detection, visual content search and digital asset management [67]

**Table 7:** Summary of data augmentation, preprocessing, and ROI handling techniques employed in KOA studies.

Category	Technique	Key Details	References
Data Augmentation	Geometric transformations	Rotation (±3°), flipping, translation, scaling	[5,94,95,104,112,124]
	Intensity-based augmentation	Brightness, contrast, gamma correction, color jitter	[5,117,124]
	Class balancing	Oversampling/stratified sampling of minority KL grades	[94,104,106,124]
	Noise injection	Gaussian noise addition	[117]
Preprocessing	Histogram equalization	Global HE or BPHE	[89,110,119,122]
	CLAHE	Clip limit ≈ 2.0, tile grid 8×8	[104,105,112,117]
	Noise filtering	Median, adaptive median, Gaussian, anisotropic filters	[104,105,107,110,122]
	Normalization	Intensity scaling; pixel spacing normalization	[90,92,94,98,106,116]
	Resizing/cropping	Fixed input size; border removal	[89,104,105,110]
	Grayscale conversion	16-bit to 8-bit grayscale (DICOM)	[92,94,110]
ROI Handling	Knee joint localization	Landmark detection using BoneFinder/FCN	[98,117,125]
	Bounding box detection	Template matching or DL-based (YOLO, Faster R-CNN)	[92,94,95,126,127]
	ROI cropping	Fixed-size patches around joint center	[89,98,105,117]
	Region proposal networks	RPN-based ROI extraction	[94,106,127]
BPHE: Brightness-Preserving Histogram Equalization; RPN: Region Proposal Network; FCN: Fully Convolutional Network.			

**Table 8:** Summary of manual segmentation techniques and annotated knee regions in KOA studies.

Reference and Year	Input Data Modality	Approach Used	Remarks
[130], 2014	X-ray	Medial, Lateral, and Minimum Joint Space Width (JSW) measured manually	Middle part of the condyles from narrowest point of joint used
[135], 2010	MRI	Cartilage segmented manually from sagittal 3D sequences; Uses endpoint segmentation software with livewire algorithm	Quality control performed by musculoskeletal radiologists
[131], 2009	X-ray	Manual joint segmentation; Software to determine joint space width boundary; Automatically identified medial subchondral bone to be used in Fractal Signature Analysis (FSA)	Six selected initialization points: tibial spine, lateral tibia, medial tibial, lateral tibial spine, medial femur, lateral femur

**Table 9:** Summary of semi-automatic segmentation techniques employed in KOA studies.

Reference and Year	Input Data Modality	Approach Used	Remarks
[133], 2017	X-ray	Random walks model for simultaneous label segmentation	Four labels: femoral, tibial, patella, background
[132], 2014	MRI	Active Shape Models (ASM) for semi-automatic segmentation	Articular cartilage segmented at distal femur
[137], 2010	MRI	Seed point within meniscus; Gaussian fit threshold; Conditional dilation; Post-processing refinement	Works for normal and degenerative menisci
[138], 2006	X-ray	Region homogeneity based on intensity; Energy function to minimize dissimilarity; Iterative mean/variance update	Manual initialization with automatic computation
[139], 2002	MRI	Semi-automated segmentation and cartilage thickness mapping	Uses 3D gradient echo MR images

**Table 10:** Summary of automatic segmentation techniques employed in KOA studies.

Reference and Year	Input Data Modality	Approach Used	Remarks
[140], 2024	MRI	Batch normalization and augmented entropy minimization; Refined using voting strategy	Uncertainty-aware pseudo supervision to boost performance
[141], 2024	MRI	Semantic segmentation of bones and cartilage; Anomaly aware segmentation	Improves bone anomaly detection
[142], 2023	X-ray	Tibia and femur segmentation using YOLOv8	OsteoGA generates images for segmentation
[5], 2022	X-ray	YOLOv3-tiny to segment ROI	Same model used for classification
[106], 2022	X-ray	Faster RCNN to detect ROI; ResNet-50 for feature extraction	RPN generates region proposals
[128], 2020	X-ray	Locate subchondral bone; Superpixel segmentation using SLIC	LBP evaluates sub-regions
[129], 2011	X-ray	Two-stage segmentation using CLAHE, template matching, and COM	Accuracy of 100%
[134], 2009	MRI	Bone statistical shape model with cartilage thickness	Femur, tibia, patella segmented

## Data Availability

No new data were created or analyzed in this study. The review used only publicly available data or published reports.
